# Combined foliar application of Zn and Fe increases grain micronutrient concentrations and alleviates water stress across diverse wheat species and ploidal levels

**DOI:** 10.1038/s41598-022-24868-1

**Published:** 2022-11-25

**Authors:** Fatemeh Shoormij, Aghafakhr Mirlohi, Ghodratollah Saeidi, Mehran Shirvani

**Affiliations:** 1 Department of Agronomy and Plant Breeding, College of Agriculture, Isfahan University of Technology, Isfahan, 84156-83111 Iran; 2 Department of Soil Science, College of Agriculture, Isfahan University of Technology, Isfahan, 84156-83111 Iran

**Keywords:** Genetics, Health care

## Abstract

This study aimed to examine the reaction of several wheat species with different ploidy levels to foliar application of zinc (Zn) and iron (Fe) under different water regimes. Thirty-five wheat genotypes, including nineteen tetraploids from ten different species, ten hexaploids from five species, and six diploids from three species, were evaluated in the field over two moisture regimes with the following four treatments: control, foliar Zn application, foliar Fe application, and foliar Zn + Fe application. The experiments were conducted according to a split-plot scheme in a randomized complete block design with two replications in each moisture regime. Water stress negatively affected all measured traits, except grain Zn and Fe content. Combined foliar application of Zn + Fe significantly increased yield and alleviated yield reduction caused by water stress. Applying Zn and Fe significantly increased both micronutrient content in grains under both moisture conditions. Tetra and hexaploid species yielded nearly four times as much grain as unimproved diploid species and were less affected by water stress. All ploidy levels responded almost similarly to Zn and Fe treatments, with the combined application being as effective as each element separately. The highest yield increase in response to combined application of Zn + Fe under the two moisture conditions and the highest grain Zn content in response to Zn application under water stress was observed in hexaploid wheat. Combined foliar application of Zn and Fe increases grain Zn and Fe and alleviates water stress's adverse effects on all wheat ploidy levels, making biofortification cost-effective.

## Introduction

Globally, wheat occupies a central place in human nutrition, and its grain yield has been continuously improved through breeding and management efforts over the past several decades^1^. However, yield improvement was associated with a substantial decline in grain protein and mineral contents, especially micronutrients such as zinc (Zn) and iron (Fe)^2,3^.

Generally, commercial wheat cultivars grain has 20–35 mg/kg Zn and Fe^4,5^, which is inadequate for human diets constituting wheat as the primary source of essential minerals^6^. Besides plant factors, almost half of cereal-cultivated soils are globally soils with inadequate concentrations of available Zn (i.e., chemically soluble Zn) further cause reducing grain Zn concentration^7^. Dietary Zn and Fe deficiency have been associated with diverse health problems such as stunted growth, anemia, depressed immune function, brain function, development impairments, and vulnerability to deadly infectious diseases due to immune dysfunction, especially in children^6^.

Among strategies to combat such deficiencies is biofortification, which increases the mineral nutrient content in crops, either by agronomic practices such as fertilization or genetic manipulation to obtain cultivars with higher mineral absorption and accumulation capabilities^8^. Crop improvement activities for Zn/Fe dense wheat focus first on exploring the available genetic diversity for Fe and Zn^6^. To identify useful variability for genetic biofortification, emphasis has been placed on screening progenitor species including *Triticum monococcum* L., *T. turgidum* L. ssp. *dicoccoides* (Körn. ex Asch. et Graebn.) Thell., *Triticum turgidum* L. ssp. *dicoccum* (Schrank) Thell., *T. aestivum* ssp. *spelta* and *Ae. tauschii*^9,10^.

In general, micronutrient fertilizers are applied to soils or sprayed onto foliage^11,12^ and effectively improve the concentration in wheat grain^4^. However, studies have shown that foliar application is more effective than soil application^13,14^. For Fe, it has been shown that Fe-EDTA appeared to be the best-applied fertilizer for increasing grain Fe concentrations^15^, mainly because Fe is quickly converted to unavailable forms when applied to soils ranging from neutral to alkaline pH, and the mobility of Fe in the phloem is poor and further reduced by endogenous chelates^16^.

In addition, abiotic stresses such as drought will likely affect the nutritional composition and wheat grain productivity which is often associated with nutrient deficiencies^17^. Zinc is involved in many physiological processes of plants, and high intrinsic Zn concentrations may help wheat tolerance to abiotic stresses. Sufficient Zn may help improve wheat tolerance to drought in different ways, including detoxifying reactive oxygen species (ROS), reducing the production of free radicals by superoxide radical-producing enzymes^18,19^, and having protective effects on photooxidative damage catalyzed by ROS in chloroplasts^20^. This suggests that the sensitivity of plants to Zn deficiency is usually more pronounced under water-limited conditions, and developing new wheat cultivars combining improved tolerance to Zn deficiency in soils and increased Zn concentration in grain is a high priority^21^.

This study examined the reaction of a broad genetic wheat base, including several species with different ploidy levels, to foliar application of Zn and Fe under two water regimes. To our knowledge, applying these combinations of treatments has not been previously reported in wheat.

## Results

### Analysis of variance

For all studied traits, the combined analysis of variance showed significant effects (P*0.01) for moisture regimes, ploidy levels, genotypes, and micronutrient treatments. Genotype × moisture regime interaction was also significant for all traits, except for FLL, FLW, grain Zn, and Fe content. The interaction effect for genotype × micronutrient application was significant for GY, NKS, and PH (Table S3). Analysis of variance for the data collected from each moisture regime also showed a significant (P*0.01) variation among genotypes, tetraploid and hexaploid species concerning all studied traits under both moisture conditions. The diploid group was significantly (P*0.01) diverse for all traits except FLL, FLW, and grain Fe content in normal moisture conditions and for KD, FLL, and grain Fe content in water stress conditions, respectively (Table S4). All traits excluding grain Zn content were significantly different, comparing 2x with each of 4x and 6x genotypes under normal water conditions.

A significant variation was observed among genotypes for all traits, except for the grain Zn and Fe content under water stress conditions. Likewise, in normal water conditions, a comparison of genotypes with tetraploid versus hexaploid levels showed a considerable difference for PH, FLW, and grain Zn content and in water stress conditions for PH, Zn, Fe content, and FLW (Table S4). Genotype × micronutrient application interaction was significant for GY, TKW, PH, and grain Zn content in normal conditions and for GY, NKS, and PH traits in water stress conditions.

### Water-stress effects on the studied traits

**Figure 1:**
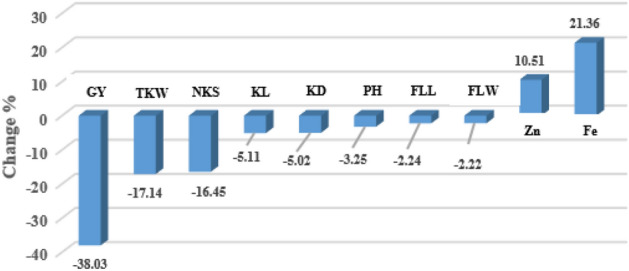
Percent change of each trait due to water stress relative to normal irrigation conditions. GY, Grain yield (g/m^2^); NKS, Number of kernel per spike; TKW, Thousand kernel weight (g); KL, Kernel length (mm); KD, Kernel diameter (mm); PH, Plant height (cm); FLL, Flag leaf length (mm); FLW, Flag leaf width (mm); Zn, Grain zinc content (µg/g); Fe, Grain iron content (µg/g).

Water stress negatively affected most measured traits, including GY, NKS, KL, KD, and PH. TKW, FLL, and FLW were also negatively affected, but their mean values were not significantly different from those in normal conditions. The highest percent reduction (38%) was observed for GY due to water stress. Contrary to other traits, grain Zn and Fe contents were significantly increased under water stress conditions, with a higher percent increase (20%) for Fe content (Table 1, Fig. 1).

**Table 1:** Mean comparison of traits under two moisture environments.

Moisture Env	GY	TKW	NKS	KL	KD	PH	FLL	FLW	Zn	Fe
Normal	721.95^a¥^	32.09^a^	107.72^a^	7.05^a^	2.79^a^	116.40^a^	183.34^a^	10.83^a^	62.56^b^	80.99^b^
Stress	447.36^b^	26.59^a^	89.79^b^	6.69^b^	2.65^b^	112.62^b^	179.23^a^	10.59^a^	69.14^a^	98.29^a^
LSD 5%	184.23	6.53	4.23	0.18	0.18	1.58	26.60	1.96	4.68	5.76

GY, Grain yield (g/m^2^); NKS, Number of kernel per spike; TKW, Thousand kernel weight (g); KL, Kernel length (mm); KD, Kernel diameter (mm); PH, Plant height (cm); FLL, Flag leaf length (mm); FLW, Flag leaf width (mm); Zn, Grain zinc content (µg/g); Fe, Grain iron content (µg/g).

^¥^For each trait, means followed by the same letter are not significantly different, using LSD test at 5% level of probability.

### Zinc and iron application effects

Under normal water conditions, Zn and Fe applications improved grain yield, but their combined application significantly increased the mean value of this trait. Water stress significantly reduced grain yield, but Zn and Fe application improved it somewhat. However, the combination of Zn + Fe application significantly alleviated yield reduction caused by water stress compared to the control moisture regime. Thousand kernel weight (TKW) was more influenced by Zn application than other treatments under both moisture conditions, with a significantly higher mean value for Zn application under water stress conditions (Table 2). Zinc + iron application enhanced NKS slightly in normal irrigation conditions. Still, under water stress situations, the Zn + Fe application significantly affected NKS compared to other treatments and eased the water stress effect to some degree. Kernel length and diameter (KL and KD) were significantly reduced due to water stress, except when Zn + Fe was applied, which lessened its effect on KL. Plant height was slightly shortened under water stress conditions, with the difference being significant only for the control in micronutrient treatments. Similarly, FLL and FLW reduction was insignificant due to water stress, with no significant differences among the micronutrient treatments (Table 2).

**Table 2:** Mean comparison of traits in response to foliar application of Zn and Fe under two moisture environments.

Moisture Env	Trait	Control	Zinc	Iron	Zinc + Iron	LSD (0.05)
Normal	GY	681.29^b¥^	726.61^ab^	709.78^b^	770.11^a^	93.19
Stress		399.96^d^	469.72^c^	434.11^cd^	485.64^c^	
Normal	TKW	31.90^ab^	32.49^a^	31.76^b^	32.24^ab^	1.96
Stress		25.75^e^	27.45^c^	26.47^d^	26.71^d^	
Normal	NKS	106.96^a^	108.69^a^	105.72^ab^	109.53^a^	11.29
Stress		86.21^c^	87.24^c^	87.67^c^	98.06^b^	
Normal	KL	6.99^a^	7.07^a^	7.04^a^	7.10^a^	0.42
Stress		6.62^b^	6.63^b^	6.65^b^	6.86^ab^	
Normal	KD	2.76^b^	2.84^a^	2.79^ab^	2.80^ab^	0.09
Stress		2.62^c^	2.59^c^	2.61^c^	2.60^c^	
Normal	PH	116.26^a^	116.43^a^	114.51^ab^	116.40^a^	2.63
Stress		110.82^c^	112.82^b^	112.08^b^	113.54^b^	
Normal	FLL	183.53^a^	188.64^a^	180^a^	181.20^a^	11.72
Stress		182.49^a^	182.29^a^	177.47^a^	174.69^a^	
Normal	FLW	10.58^a^	10.84^a^	10.77^a^	11.17^a^	0.67
Stress		10.65^a^	10.79^a^	10.59^a^	10.33^a^	
Normal	Zn	54.98^c^	68.36^ab^	60.25^bc^	66.63^ab^	11.59
Stress		62.56^bc^	75.41^a^	68.92^ab^	69.68^ab^	
Normal	Fe	74.86^d^	79.79^d^	89.57^c^	79.72^d^	11.04
Stress		91.81^bc^	92.61^bc^	111.25^a^	97.47^b^	

GY, Grain yield (g/m^2^); NKS, Number of kernel per spike; TKW, Thousand kernel weight (g); KL, Kernel length (mm); KD, Kernel diameter (mm); PH, Plant height (cm); FLL, Flag leaf length (mm); FLW, Flag leaf width (mm); Zn, Grain zinc content (µg/g); Fe, Grain iron content (µg/g).

^¥^For each trait, means followed by the same letter are not significantly different, using LSD test at 5% level of probability.

Contrary to other traits, grain Zn and Fe contents were elevated under water stress compared to normal irrigation conditions. As expected, at water stress and non-stress conditions, the highest grain Zn and Fe content was observed for Zn and Fe application treatment, respectively, with their values being higher at water stress conditions (Table 2).

### Response of wheat ploidy levels to water stress and foliar application of zinc and iron

Grain yield was nearly four times higher in tetra and hexaploid species than in diploids under normal moisture conditions. Water stress reduced grain yield to a different extent at different micronutrient treatments for all wheat ploidy levels (2x, 4x, and 6x) (Table 3). A higher yield reduction was observed in 2x, followed by 4x, and finally 6x  species at control treatment of micronutrient application. For 2x  and 4x  species, Zn and its combination with Fe applications slightly increased grain yield under normal irrigation conditions and eased yield reduction under water stress. However, the mean yields for 6x species were significantly increased in both moisture conditions at Zn and Zn + Fe applications (Table 3).

**Table 3:** Mean comparison of traits for different wheat ploidy levels in response to foliar application of Zn and Fe under two moisture environments.

Moisture Env	Trait	PL^€^	Control	Change (%)	Zn	Change (%)	Fe	Change (%)	Zn + Fe	Change (%)	LSD (0.05)
Normal	GY	2x	212.71^hij¥^	− 48.09^δ^	210.83^hij^	− 26.78	273.12^h^	− 51.95	253.54^hi^	− 33.61	53.80
Stress			110.41^k^		154.37^jk^		131.25^jk^		168.33^ijk^		
Normal		4x	834.53^c^	− 41.93	824.01^cd^	− 37.22	868.35^c^	− 46.09	847.17^c^	− 40.14	
Stress			484.60^g^		517.30^fg^		468.09^g^		507.10^g^		
Normal		6x	996.62^b^	− 39.31	1145^a^	− 35.59	987.87^b^	− 28.84	1209.62^a^	− 35.39	
Stress			604.87^f^		737.50^de^		703^e^		781.50^cde^		
Normal	TKW	2x	13.32^gh^	− 7.96	15.83^f^	− 10.80	15.87^f^	− 16.45	15^fg^	− 9.07	1.13
Stress			12.26^h^		14.12^fgh^		13.26^gh^		13.64^gh^		
Normal		4x	39.58^c^	− 17.21	40.32^bc^	− 17.58	39.83^c^	− 17.98	39.49^c^	− 18.06	
Stress			32.77^e^		33.23^de^		32.67^e^		32.36^e^		
Normal		6x	42.09^ab^	− 23.45	41.34^abc^	− 15.31	39.58^c^	− 15.41	42.22^ab^	− 19.16	
Stress			32.22^e^		35.01^d^		33.48^de^		34.13^de^		
Normal	NKS	2x	103.92^bcd^	− 20.61	103.58^bcd^	− 15.21	97.42^def^	− 10.18	103.92^bcd^	− 13.08	6.52
Stress			82.50^g^		87.83^fg^		87.50^gf^		90.33^efg^		
Normal		4x	101.32^cde^	− 15.72	105.95^a–d^	− 17.39	107.34^a–d^	− 20.35	113.68^ab^	− 14.14	
Stress			85.39^g^		87.53^fg^		85.50^g^		97.61^def^		
Normal		6x	115.65^a^	− 21.53	116.55^a^	− 25.91	112.40^abc^	− 19.93	111^abc^	− 4.28	
Stress			90.75^efg^		86.35^ fg^		90^gf^		106.25^a–d^		
Normal	KL	2x	6.95^c–g^	− 9.06	7^b–f^	− 10.43	7.01^b–f^	− 7.99	6.96^b–f^	− 3.88	0.24
Stress			6.32^ij^		6.27^j^		6.45^ghi^		6.69^g–j^		
Normal		4x	7.30^abc^	− 3.84	7.40^ab^	− 5.27	7.28^a–d^	− 3.71	7.47^a^	− 3.35	
Stress			7.02^e–g^		7.01^b–e^		7.01^b–f^		7.22^a–e^		
Normal		6x	6.73^f–i^	− 3.27	6.82^e–h^	− 2.79	6.85^d–h^	− 5.26	6.88^b–h^	− 2.91	
Stress			6.51^g–j^		6.63^f–i^		6.49^hij^		6.68^f–j^		
Normal	KD	2x	2.26^f^	− 1.32	2.40^e^	− 5.42	2.35^ef^	− 4.68	2.29^ef^	− 1.31	0.05
Stress			2.23^f^		2.27^f^		2.24^f^		2.26^f^		
Normal		4x	2.90^bc^	− 6.21	2.96^ab^	− 9.80	2.87^bc^	− 5.92	2.94^bc^	− 9.18	
Stress			2.72^d^		2.67^d^		2.70^d^		2.67^d^		
Normal		6x	3.13^a^	− 8.31	3.16^a^	− 10.13	3.15^a^	− 8.25	3.18^a^	− 9.43	
Stress			2.87^bc^		2.84^c^		2.89^bc^		2.88^bc^		
Normal	PH	2x	115^def^	− 3.52	114.39^efg^	− 1.24	114.98^d–g^	− 3.03	114.97^d–g^	− 2.17	1.51
Stress			110.95^d–g^		112.97^fgh^		111.50^hIj^		112.48^def^		
Normal		4x	117.13^bcd^	− 4.85	118.74^ab^	− 2.28	115.88^cde^	− 0.85	118.35^bc^	− 2.50	
Stress			111.45^hij^		116.03^cde^		114.90^d–g^		115.39^def^		
Normal		6x	116.65^b–e^	− 5.94	116.17^b–e^	− 5.77	112.66^gh^	− 2.49	115.89^fgh^	− 2.70	
Stress			109.72^j^		109.47^j^		109.85^ij^		112.76		
Normal	FLL	2x	118.19^g^	− 3.86	131.26^f^	− 10.12	117.68^g^	0.02	122.28^def^	− 7.59	6.76
Stress			113.63^g^		117.98^g^		117.70^g^		113^g^		
Normal		4x	228.58^a^	− 3.92	220.27^abc^	1.15	214.40^bcd^	− 1.33	207.85^ed^	1.04	
Stress			219.61^abc^		222.80^a,b^		211.54^d–e^		210.01^def^		
Normal		6x	212.78^bcd^	− 3.53	211.88^b–e^	− 1.54	210.79^cde^	− 4.97	211.30^fgh^	− 3.82	
Stress			205.26^ed^		208.62^cde^		200.31^e^		203.22^ed^		
Normal	FLW	2x	6.33^h^	− 1.42	5.74^h^	3.83	6.06^gh^	6.44	6.30^gh^	− 3.81	0.38
Stress			6.24^gh^		5.96^gh^		6.45^g^		6.06^gh^		
Normal		4x	12.84^ed^	0.93	13.14^b–e^	− 0.53	13.15^b–e^	− 3.35	13.37^a–d^	− 9.35	
Stress			12.96^ed^		13.07^ab^		12.71^def^		12.12^f^		
Normal		6x	12.48^ef^	3.04	13.62^abc^	− 8.52	13.11^cde^	− 3.74	13.85^a^	− 7.58	
Stress			12.86^ed^		12.46^ef^		12.62^ef^		12.80^def^		
Normal	Zn	2x	61.33^e–h^	11.23	74.53^abc^	3.73	61.30^e–h^	23.28	73.81^a–d^	− 0.22	6.69
Stress			68.22^a–f^		77.31^ab^		75.57^ab^		73.65^a–d^		
Normal		4x	52.69^gh^	12.09	70.04^a–e^	11.95	61.41^e–h^	12.95	63.61^c–g^	9.06	
Stress			59.06^e–h^		78.41^a^		69.36^a–f^		69.37^a–f^		
Normal		6x	50.92^h^	18.64	60.55^e–h^	16.43	58.06^fgh^	6.48	62.46^d–h^	5.72	
Stress			60.41^e–h^		70.50^a–e^		61.82^e–h^		66.03^c–f^		
Normal	Fe	2x	82.70^f–k^	18.85	85.27^f–i^	16.68	99.41^cde^	17.75	89.98^efg^	17.25	6.37
Stress			98.29^cde^		99.49^cde^		117.06^a^		105.50^bc^		
Normal		4x	68.07^ l^	26.15	74.82^i–l^	18.39	84.46^f–k^	20.86	75.27^h–l^	24.46	
Stress			85.87^fgh^		88.58^fgh^		102.08^cd^		93.68^def^		
Normal		6x	73.80^kl^	23.67	79.27^g–k^	13.26	84.85^f–j^	35.09	73.93^jkl^	26.09	
Stress			91.27^def^		89.78^efg^		114.62^ab^		93.22^def^		

GY, Grain yield (g/m^2^); NKS, Number of kernel per spike; TKW, Thousand kernel weight (g); KL, Kernel length (mm); KD, Kernel diameter (mm); PH, Plant height (cm); FLL, Flag leaf length (mm); FLW, Flag leaf width (mm); Zn, Grain zinc content (µg/g); Fe, Grain iron content (µg/g).

^¥^For each trait, means followed by the same letter are not significantly different, using LSD test at 5% level of probability.

^δ^Change by water stress relative to normal irrigation conditions.

^€^Ploidy level.

Similar to grain yield, TKW was about four times higher in 4x and 6x species than the 2x ones under normal moisture conditions. Water stress reduced TKW in all ploidy level species, with 2x being less affected. At this moisture condition, the lowest TKW mean was observed for the control treatment and the highest for Zn application. A comparable NKS was observed for all wheat ploidy levels at normal water conditions. Water stress significantly reduced NKS for all three ploidy level species, similarly. The lowest reduction of this trait due to water stress was observed for foliar application of Zn + Fe in hexaploid species. The highest KL belonged to 4x species, with the means for 2x and 6x genotypes being almost comparable under normal water conditions. The mean KL was reduced under water stress conditions for all ploidy levels, with 2x genotypes were more affected. Foliar application of Zn + Fe abridged the effects of water stress on KL at all ploidy levels to some degree (Table 3).

In contrast to KL, 2x and 4x species had the lowest mean KD under normal moisture conditions, and 6x genotypes had the highest. At the same time, water stress influenced 6x species more than 2x, and 4x with percent KD reduction higher for hexaploids. Zinc, Fe, and their combined applications had no significant effect on this trait. The mean plant height (PH) was almost the same for all wheat ploidy levels under normal water conditions and was not significantly influenced by water stress. The treatments also had no significant effect on this trait. The mean values of FLL and FLW were almost two times higher for 4x and 6x than 2x genotypes under normal water conditions. Compared to other traits, the reduction rate of these two traits was non to minimal under water stress conditions. A meaningful pattern of change due to micronutrient treatments was not observed for these two traits. Compared to 4x and 6x species, the grain Zn and Fe content were higher for 2x genotypes under normal moisture conditions (Table 3). Unlike other traits, the grain Zn and Fe contents were increased under stress conditions regardless of the micronutrient treatments. However, Zn and Zn + Fe treatments significantly increased grain Zn content, with the mean value being highest at Zn application under water stress conditions. Correspondingly, the most elevated content of grain Fe was observed for Fe application, and the highest value was observed for this treatment under water stress conditions (Table 3).

### Species discrimination based on biplot analysis for micronutrient treatments

#### Control treatment

**Figure 2:**
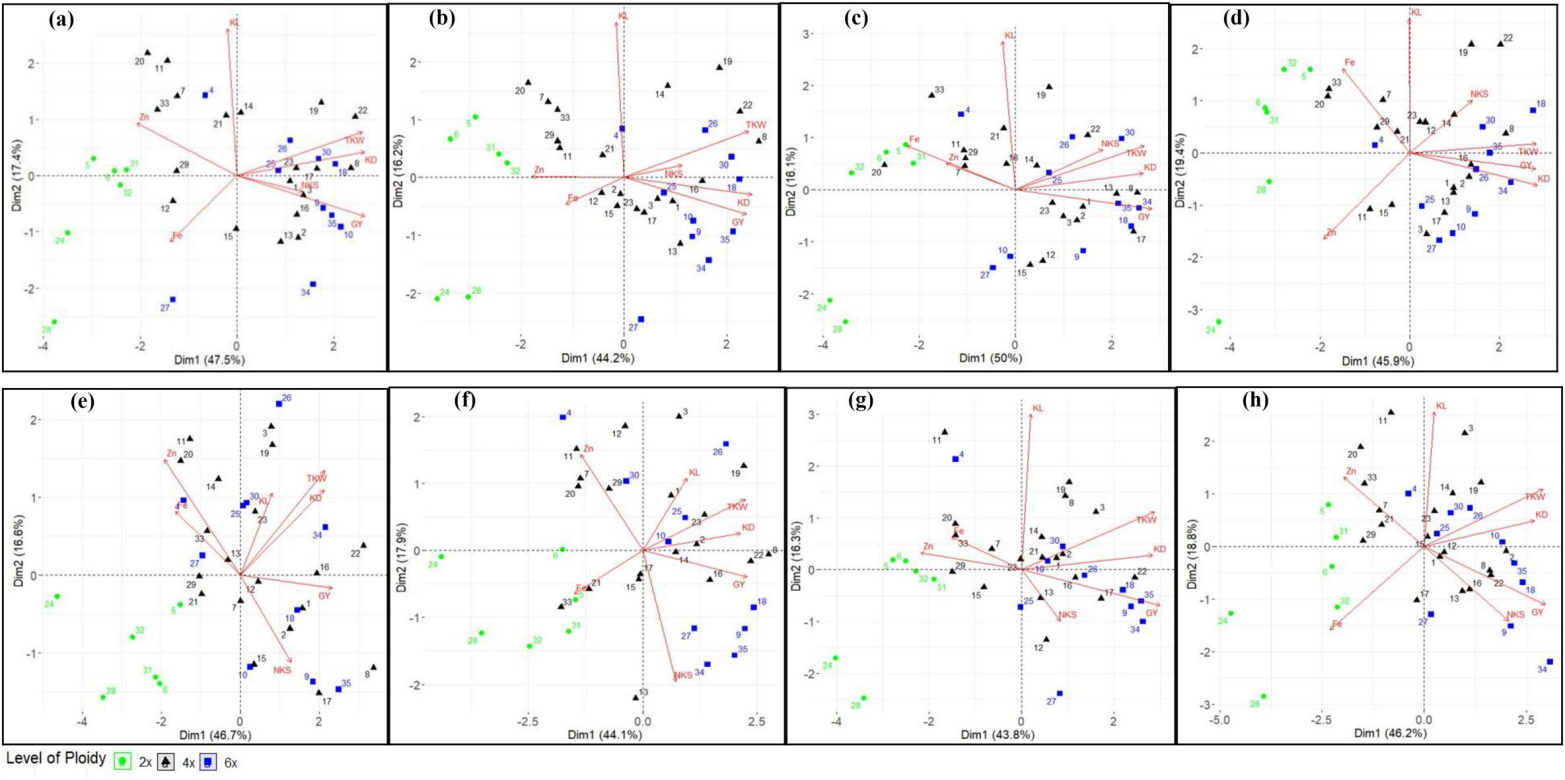
Visualization of biplot based on PCA and interrelationships between traits for 35 genotypes with different ploidy levels/species subjected to four treatments under normal irrigation (a = control, b = zinc, c = iron, and d = zinc + iron applications) and water stress (e = control, f = zinc, g = iron, and h = zinc + iron applications). *Abbreviations*; Grain yield (GY g/m^2^), Number of kernel per spike (NKS), Thousand kernel weight (TKW g), Kernel length (KL mm), Kernel diameter (KD mm), Grain zinc content (Zn µg/g), and Grain iron content (Fe µg/g).

Under normal irrigation, in the control treatment (Fig. 2a) principal components PC1 and PC2 explained 47.5 and 17.4% of the variation, respectively. The PC1 was positively associated with GY, KD, and TKW, and negatively correlated with Zn. PC2 was positively loaded with KL. Most emmer genotypes (numbers 20, 33, 7, 21, and 29) and *T. polonicum* (#11) along with one spelt genotype (#4) were placed in a distinct group from other genotypes in the left quadrant of the biplot with a high value of PC2 and a low value of PC1. Wild einkorn species included *T. urartu* (#5 and 6) and *T. boeoticum* (#31 and 32) were placed in one group distinct from einkorn *T. monoccocum* (#24 and 28) and were located in the left quadrant of the biplot with a medium value of PC2 and low value of PC1. A high value of PC1 and a low value of PC2 on the right quadrant of the biplot was observed for the improved tetra and hexaploid genotypes. For both PCs, *T. turanicum* (#19 and 22) had a high value.

Under water stress (Fig. 2e), PC1 and PC2 justified 46.7 and 16.6% of the variation, respectively. The PC1 was positively associated with GY, KD, and TKW, and negatively correlated with Zn and Fe. PC2 was positively loaded with NKS. A high value of PC2 and low to medium PC1 were observed for genotypes 11, 20, 14, 3, 19, and 26. Among them, *T. petropavlovski* (#26) revealed the highest PC2, whereas species *T. ispahanicum* (#20 and 11) showed higher grain Zn content, and a spelt genotype (#4) showed higher grain Fe content. PC1 was highest for *T. durum* (#8) and *T. turanicum* (#22) with a low to medium PC2. Compared with 4x and 6x, diploid species showed a greater decline in grain yield, especially *T. monococcum*. Grain Zn and Fe content were increased due to water stress especially, for genotypes 34, 26, 25, 30, and 3. In normal and water stress conditions, 2x were separated from 4x and 6x on the left side of the biplot chart, while on the right side, the 4x and 6x did not detach totally.

### Zinc treatment

In Zn application, 44.2 and 16.2% of variations were justified by PC1 and PC2 under normal irrigation (Fig. 2b), respectively. The PC1 was positively associated with KD, GY, and TKW, and negatively correlated with Zn. PC2 was positively loaded with KL. The majority of the emmer genotypes (numbers 20, 7, 33, 29, and 21), one *T. polonicum* (#11), one spelt genotype (#4), and wild einkorns (#31, 32, 5, and 6) were placed in a distinct group from other genotypes in the left quadrant of the biplot with a high value of PC2 and low to a medium value of PC1. In addition, *T*. *boeoticum* (#31 and 32) showed higher scores for grain Zn. Also, synthetic cross (#35) exhibited higher scores for GY. PC1 was highest for *T. durum* (#8) and *T. turanicum* (#22, 19), with a high value of PC2, and exhibited higher scores for TKW.

Under water stress (Fig. 2f), PC1 and PC2 showed 44.1 and 17.9% variations. The PC1 was correlated positively with GY, TKW, and KD. PC2 was positively associated with Zn and Kl, and negatively correlated with NKS. Water stress resulted in the separation of genotypes 7, 20, 29, 4, 30, 11, and 12 in a group with a high value of PC2 and a low value of PC1 on the left quadrant of the biplot. Emmer genotypes (#21 and 33) and *T. urartu* (#5) showed a higher score for grain Fe. Grain Zn was increased due to water stress, especially for genotypes 3, 4, 26, 30, and 10. Among the diploid species, two genotypes of *T. monococcum* showed an increase in PC2 compared to the normal irrigation conditions, particularly genotype 24. PC1 was highest for genotypes 22 and 8, with a medium value of PC2. PC2 was highest for genotypes 4, 1, and 3 with a low to medium value of PC1. Across both water conditions and Zn application diploids separated from tetra and hexaploids on the left side of the biplot chart, while tetra and hexaploids species did not completely detach on the right side.

### Iron treatment

Under normal irrigation and Fe treatment (Fig. 2c), PC1 and PC2 described 50 and 16.1% of the variation, respectively. The PC1 was positively associated with GY, TKW, and KD, and negatively loaded with Fe and Zn. PC2 was positively loaded with KL. The position of the genotypes in the left quadrants of the biplot with a high value of PC2 was the same as with Zn treatment under normal conditions. Furthermore, genotypes 5 and 31 showed higher scores for grain Fe, and likewise, genotypes 7, 29, and 11 displayed higher scores for grain Zn. Synthetic crosses (#34 and 35) and *T. aestivum* (#18) exhibited higher scores for GY. PC1 was highest for genotypes 34 and 8 with a medium value of PC2. PC2 was highest for *T. dicoccoides* (#33) and 19, with a low to medium value of PC1.

Under water stress conditions (Fig. 2g), PC1 and PC2 presented 43.8 and 16.3% of the variation, respectively. The PC1 was positively associated with GY, TKW, and KD, and negatively loaded with Zn and Fe. PC2 was positively loaded with KL. Several emmer genotypes (#20, 33, 7, and 29), along with genotypes number 4, and 11 were grouped together with a high value of PC2 and low value of PC1 on the left quadrants of the biplot. Among them, genotypes 11 and 4 had the highest value of PC2 and low value of PC1. Moreover, emmer genotypes 20 and 33 showed the highest score for grain Fe whereas *T. urartu* genotypes 5 and 6 presented a high score for grain Zn. Synthetic crosses (#35 and 34) and Chines spring (#9) registered the highest score for GY and a low value of PC2. Genotypes 19, 8, and 3 revealed a high value for both PCs.

### Zinc + Iron treatment

Under normal irrigation and Zn + Fe treatment (Fig. 2d), PC1 and PC2 described 45.9 and 19.4% of the variation, respectively. The PC1 was positively associated with KD, TKW, and GY and negatively associated with grain Zn. PC2 was positively correlated with KL and Fe. In this condition, the position of genotypes on the left quadrants of the biplot with high PC2 was similar to Zn and Fe treatment under normal irrigation conditions, except for genotype 11. Emmer genotypes 33 and 20 showed a high score for grain Fe. Genotype 18 had the highest PC1 value and a high PC2. The highest PC2 and a high value of PC1 belonged to *T. turanicum*.

Under water stress, (Fig. 2h), PC1 and PC2 described 46.2 and 18.8% of the variation, respectively. The PC1 was positively associated with GY, TKW, and KD, and negatively associated with Zn and Fe. PC2 was positively correlated with KL. Similar to non-stress conditions, mostly the same genotypes were grouped in the left quadrants of the biplot with high PC2 and low to medium PC1. Genotype 18 showed a high score for GY. Genotypes 20 and 33 showed top scorers for grain Zn while genotype 32 had a high value for grain Fe. Synthetic cross (#34) had the highest PC1 with a low value of PC2, and genotype 11 had the highest PC2 with a low value of PC1. Water stress increased PC1 and PC2 values for *T. compactum* (#10) compared to normal conditions. Congruent with other treatments, the diploids were separated from tetra and hexaploids on the left side of the biplot chart under both water conditions.

## Discussion

Contrary to all measured traits, grain Zn and Fe contents (µg/g) were increased under water-limited conditions. A concentration effect has been suggested due to the smaller grain size and a higher ratio of aleurone to endosperm under moisture stress conditions for this change^22^. Besides grain yield and its components, a significant reduction in KL and KD, which are directly related to grain size, was recorded under water-limited conditions compared to the well-water situation.

Zinc and Fe are needed to regulate many cellular processes in plants like chlorophyll synthesis, and their shortages lead to decreased cellular metabolism, affecting plant yield. Soil applications of Zn and Fe can improve grain yield, but some studies have shown that foliar application is more effective than soil application^13,14^. Under water-limited conditions, soil application may be ineffective as the transport of Zn to plant roots predominantly occurs via diffusion, and moisture provides a physical medium for an effective diffusive flux of Zn^23^. In the present study, foliar Zn and Fe applications under normal water conditions improved grain yield to some extent, but their combined application significantly increased grain yield compared to control. Also, at water stress condition, a significantly higher GY was observed for Zn and Zn + Fe treatments and alleviated yield reduction caused by water stress compared to the control treatment. It has been noted that micronutrients such as Zn can mitigate adverse effects of water stress through regulation of plant processes, including up-regulation of abscisic acid to optimize stomatal closure, higher production, and activity of anti-oxidative enzymes, and upregulation of genes involved in water stress^24–26^. It seems that among the three yield component traits measured, TKW and KL had more contribution to higher grain yield than KD as the formers were less reduced due to water stress, especially at Zn + Fe application than KD (Table 2).

In addition to the nutrient concentration effect due to the smaller grain size under moisture stress conditions, Zn and Fe applications significantly increased the content of these micronutrients in grain. These results strongly support previous findings suggesting the efficiency of Zn and Fe foliar application in raising the grain Zn and Fe content, especially under water stress conditions, where soil application may limit their mobility and plant availability^27,28^. It is also believed that due to the poor mobility of Fe in the phloem, Fe fertilization appears to be less effective than Zn fertilization in the enrichment of cereal grains^4,7^. However, this was not the case in our research, and Fe foliar application was even more effective in increasing the grain Fe content than the Zn application and grain Zn content (Table 2). The timing of foliar application is also a determining factor in increasing grain Zn and Fe content, with the late growth stages being the best^7^. In this research, Zn and Fe were foliarly applied at two late plant growth stages of booting and milky-dough.

Foliar application of Zn and Fe positively affected all traits except PH, FLL, and FLW, particularly in water-limited conditions, where the stress effect on traits was mitigated to some degree. Zinc + iron treatment increased grain yield and its components and Zn application improved NKS and TKW under both moisture conditions. Our finding was consistent with previous studies in this respect^28,29^.

Our results regarding the response of different wheat ploidy levels (2x, 4x, and 6x) to water stress and different micronutrients application were consistent with other reports^30^. In this study, 4x and 6x species yielded nearly four times as much grain as 2x species at both moisture regimes. Diploid species are generally less developed than 4x and 6x species that have undergone many selection cycles to increase grain yield^31^. This is evident in several superior attributes of 4x and 6x species, including *T. aestivum*, and *T. durum* compared to diploids, *T. boeoticum*, and *T. urartu*. Also, we found much lower values for TKW and GY for diploids, reflected by their smaller KL and KD, compared to higher wheat ploidy levels.

The impact of water stress on grain yield varied by ploidy levels (2x, 4x, and 6x), with a higher reduction (48%) observed for 2x at the control treatment of micronutrient application. All wheat ploidy levels responded almost similarly to Zn, Fe, and Zn + Fe treatments, resulting in an increased yield under normal moisture conditions or a lower reduction at water stress. At the same time, the application of Zn, Fe, and their combination significantly increased the concentration of these micronutrients in grain which is in agreement with previous reports^32,33^. This suggests that Zn and Fe foliar applications can simultaneously increase grain yield and Zn and Fe content under both normal and water-stressed conditions.

The combined application of Zn and Fe was as practical as the application of each element separately, which may make Zn and Fe biofortification cost-efficient. Interestingly, the highest grain yield increase in response to the combined application of Zn and Fe under the two moisture conditions and the highest grain Zn content in response to foliar application of Zn under water stress conditions was observed in 6x wheat. Hexaploid wheat (*Triticum aestivum* L*.*) accounts for around 95% of the world's wheat production and is a staple crop for an estimated 40% of the world’s population^34^. Most modern wheat cultivars are inherently low in grain Zn and Fe, a severe human health concern.

Principal component analysis depicted in biplot charts (Fig. 2a–h) revealed discrimination of species in response to micronutrient treatments. Diploid species of *T. boeoticum, T. monoccocum,* and *T. urartu* are considered wild or domesticated wild^35^. As expected, these unimproved species reacted inconsistently to micronutrient treatments compared to more improved 4x and 6x counterparts. At both irrigation conditions and all micronutrient treatments, 2x had a much lower yield than 4x and 6x species. Compared to modern 4x and 6x cultivars, they grouped away on the left quadrants of the biplot, exhibiting higher grain Zn and Fe content.

A total of ten species were studied at the 4x level. In both irrigation conditions and all treatments, mostly wheat landraces belonging to *T. dicoccoides* (wild emmer), *T. dicoccum* (emmer), *T. ispahanicum* (emmer), and one *T. polonium* genotype (#11) were separated from more improved species and mainly occupied the left quadrants on the biplot chart linked with higher grain Zn, Fe, and KL and lower GY, TKW, NKS, and KD. In contrast, the improved genotypes of tetraploids that are naturally poor in Zn and Fe were placed on the right quadrants of the biplot associated with low grain Zn and Fe, and higher GY, TKW, KD, and NKS. This points to the known fact that the landrace genotypes cannot compete with the modern bred varieties for yield, but they have many valuable characteristics, such as higher grain Zn and Fe content^19^. Among the kinds of wheat tested so far, wild emmer, *Triticum turgidum* ssp. *dicoccoides* contain the highest concentrations of grain Zn and Fe^5^. The *Gpc-B1* allele located on chromosome six of this species has been well characterized and associated with higher grain protein, Zn, and Fe^36^.

Hexaploids consisted of five species with different degrees of improvement. In both moisture conditions and all treatments, spelt genotype number 4 was placed in the left quadrant of the biplot, close to emmer and einkorn genotypes, and associated with higher grain Zn and Fe, and lower GY, TKW, NKS, and KD. Reports^37,38^ indicate that among hexaploid wheat, *T. spelta*, despite having lower grain yield, has higher grain protein, Zn, and Fe. On the other hand, the majority of genotypes of improved species, including synthetic crosses and *T. aestivum*, were found in the right quadrants in both moisture conditions and all treatments. These genotypes had high GY, TKW, NKS, KD, and low grain Zn, Fe, and KL. This may further confirm that yield improvement was associated with a substantial decline in grain mineral contents^2,3^ and, therefore, the better response of these genotypes to micronutrients application such as Zn and Fe.

Ploidy levels and species within each ploidy level responded differently to water stress concerning yield reduction, with 2x being more affected and becoming farther apart from 4x and 6x species mainly on the lower left quadrant of the biplot chart in all treatments. Even though 6x genotypes had higher yield and lower grain Zn and Fe than 4x genotypes under normal conditions, they showed a more pronounced decrease in yield under water stress. In general, 4x wheat is more tolerant to water stress than the 6x counterparts^39^. As a result, in most treatments and both moisture conditions, the 4x species of *T. turanicum* and *T. durum* (#8) showed high GY, TKW, and KD and grain Zn and Fe.

## Conclusion

Water stress negatively affected all measured traits except for grain Zn and Fe content, which was increased under water-limited conditions. Under normal and water stress conditions, foliar Zn and Fe applications somewhat improved grain yield, but their combined application significantly increased this trait compared to the control treatment. Application of Zn + Fe also alleviated grain yield reduction caused by water stress. In addition to the concentration effect due to the smaller grain size under moisture stress conditions, Zn and Fe applications significantly increased the content of these two micronutrients in grain. Consistent with other reports, our results showed that 4x and 6x species yielded nearly four times as much grain as unimproved 2x species at both moisture levels. The negative impact of water stress on grain productivity varied by ploidy levels, with a higher grain yield reduction observed in 2x wheat. All ploidy levels responded almost similarly to Zn, Fe, and Zn + Fe foliar fertilization. The combined application of Zn + Fe was as practical as each element separately, making Zn and Fe biofortification cost-effective. Interestingly, the highest increase of grain yield in response to combined application of Zn + Fe under both moisture conditions and the highest grain Zn content in response to foliar application of Zn under water stress conditions was observed in 6x wheat.

Consistent with the above findings, PCA analysis categorized different species in response to micronutrient treatments. At both water conditions, wheat landraces and wild species were separated on the biplot chart from high-yielding improved genotypes of 4x and 6x species. The less improved landraces naturally contain higher grain Zn and Fe since they have not been selected aginst in the process of yield improvement which is the case for most varieties of durum and bread wheat.

## Materials and methods

### Plant Materials and experimental method

In a preliminary study, a collection of 144 wheat genotypes consisting of different ploidy levels with a wide geographical distribution were tested for yield, and 86 higher yielding genotypes were selected for grain Zn and Fe measurements. Again, considering all ploidy levels and geographical origin, 35 genotypes with higher grain Zn and Fe concentrations were designated for the current study (data not shown). The collection comprised ten hexaploid genotypes from five species, 19 tetraploids from 10 species, and six diploids from three species (Table S1). A split-plot scheme in a randomized complete block design with two replication was used in each of two irrigation regimes. The experiment was conducted at the Research Farm of Isfahan University of Technology (IUT) (32°32′ N and 51°23′ E, with 1630 m altitude) during the 2019–2020 growing seasons. Each plot consisted of two rows, each 100 cm long, 20 cm apart (0.40 m^2^), and 1.25 cm plant spacing. The micronutrient application was considered as the main factor and the genotype as sub factor. The soil in the experiment site was clay loam with a pH of 7.5 (Table S2) and its average annual precipitation and temperature were 140 mm and 15 °C, respectively. All plots were uniformly irrigated until 50% of all genotypes were at anthesis. At this stage, two irrigation regimes were applied to the experimental plots using a volumetric counter and polyethylene pipes from a pumping station. To provide non-stress and water stress conditions, irrigation was applied when 40 and 80% of the total available water around the root zone were depleted, respectively^40^.

The foliar application of Fe and Zn was performed at two plant growth stages of booting (scale 40) and early milk stage (scale 73)^41^ suggested to be the most responsive stages^7^. Fe-EDTA and Zn-EDTA were applied by spraying a 0.25% (w/v) solution containing 200 mg L^−1^ of Tween 20 as a surfactant^42^. The 0.25% w/v (2.5 g L^−1^) solutions of Zn-EDTA and Fe-EDTA were prepared by dissolving appropriate amounts of Fe-EDTA (13.2% Fe) or Zn-EDTA (14.0% Zn) chelates (supplied by the TradeCorp AZ®) in 250-L polyethylene tank containing 220 L tap water.

An equivalent amount of water was sprayed on plants in control plots. For Zn + Fe treatment, the Zn-EDTA was sprayed first, and three days later, the Fe-EDTA was applied. All sprayings were conducted to cover the leaf surface uniformly at the point of runoff late in the afternoon, considering the wind velocity. Except for irrigation and foliar treatments, all other field operations were done uniformly for all experimental plots. Our plant material complies with relevant institutional, national, and international guidelines and legislation.

### Measurement of the traits and Data analysis

The traits, including the number of kernel per spike (NKS), plant height (PH cm), flag leaf length (FLL mm), and flag leaf width (FLW mm), were measured on five samples randomly selected from each plot. The PH, FLL, and FLW were measured at late anthesis. Grain yield per plot (GY g/m^2^) was the total grain weight harvested from each plot. Kernel length and diameter (KL, KD mm) were measured on 100 grains using a caliper and then averaged. One hundred grains were counted, weighed, and used for the calculation of thousand kernel weight (TKW g). The grain Zn and Fe concentrations were determined as follows. A grain sample (0.2 g) from each plot was ashed at 550 °C for three h. Inorganic ions were then extracted using 10 ml 2 N HCl, and the volume of each sample was standardized to 100 ml. The samples were analyzed using a Rayleigh, WFX-210 flame atomic absorption spectrometer. The detection limits for Fe and Zn were 0.01 and 0.007 mg L^−1^, respectively. All grain micronutrient concentrations were expressed on a dry weight basis.

Before analysis of variance (ANOVA), the Kolmogorov–Smirnov test was conducted to examine the normality distribution of data. The Bartlett’s test was used to test the homogeneity of residual variances. Subsequently, the differences among genotypes, between irrigation regimes, and their interactions were examined in a combined analysis of variance, using SAS release 9.4 (SAS Institute, Cary, NC, USA). Principal component analysis (PCA) was performed to reduce the multiple dimensions of data space and also used to identify the relationships between traits and dispersion of the treatments using the R package software^43^.

## Supplementary Information

Supplementary Tables.

## Data Availability

The datasets used and/or analyzed during the current study are available from the corresponding author on reasonable request.
