# Supplementary material for: Combined foliar application of Zn and Fe increases grain micronutrient concentrations and alleviates water stress across diverse wheat species and ploidal levels
Source: Sci Rep. 2022 Nov 25;12:20328. doi: 10.1038/s41598-022-24868-1 (PMC9700663; doi:10.1038/s41598-022-24868-1)
Supplement: Supplementary file 1 — Supplementary Tables. [file 41598_2022_24868_MOESM1_ESM.docx]

| **Species** | **Genotype Number** | **Ploidy levels** | **Genotype**  **code** | **Species** | **Genotype Number** | **Ploidy levels** | **Genotype**  **code** | **Species** | **Genotype Number** | **Ploidy levels** | **Genotype**  **code** |
| --- | --- | --- | --- | --- | --- | --- | --- | --- | --- | --- | --- |
| *T. monococcum* | 24 | 2x | IPK^1^,TRI 565 | *T. persicum* | 12 | 4x | IPK, TRI 9535 | *T. dicoccoides* | 33 | 4x | Iran |
| *T. monococcum* | 28 | 2x | Iran | *T. persicum* | 15 | 4x | Iran | *T. aestivum* (Chines Spring) | 9 | 6x | Iran |
| *T. urartu* | 5 | 2x | Iran | *T. turanicum* | 22 | 4x | IPK,TRI 17606 | *T. aestivum* | 18 | 6x | Iran |
| *T. urartu* | 6 | 2x | Iran | *T. turanicum* | 19 | 4x | Iran | *T. compactum* | 10 | 6x | Iran |
| *T. boeoticum* | 31 | 2x | Iran | *T. aethiopicum* | 2 | 4x | IPK, TRI 15657 | *T. sphaerococcum* | 27 | 6x | IPK,TRI 18664 |
| *T. boeoticum* | 32 | 2x | Iran | *T. aethiopicum* | 3 | 4x | IPK, TRI 15593 | Synthetic cross^4^ | 34 | 6x | CIMMYT,BW 39452 |
| *T. turgidum* | 1 | 4x | Iran | *T. timopheevii* | 23 | 4x | IPK, TRI 7301 | Synthetic cross | 35 | 6x | CIMMYT, BW 31696 |
| *T. turgidum* | 13 | 4x | IPK, TRI9652 | *T. ispahanicum* | 20 | 4x | IPK,TRI 6177 | *T. petropavlovski* | 26 | 6x | IPK,TRI 12908 |
| *T. durum* | 8 | 4x | ICARDA^2^ | *T. dicoccum* | 14 | 4x | IPK,TRI 19124 | *T. spelta* | 9 | 6x | IPK,TRI 3429 |
| *T. durum* (Langdon) | 16 | 4x | Iran | *T. dicoccum* | 29 | 4x | CIMMYT^3^, 49655 | *T. spelta* | 30 | 6x | Iran |
| *T. polonicum* | 17 | 4x | Iran | *T. dicoccum* | 7 | 4x | Iran | *T. vavilovii* | 25 | 6x | IPK, TRI 11554 |
| *T. polonicum* | 11 | 4x | IPK, TRI 1896 | *T. dicoccum* | 21 | 4x | IPK,TRI 17619 |  |  |  |  |

**Table S1 Information on 35 wheat genotypes with different ploidy levels and species used in the study**

^1^ Leibniz Institute of Plant Genetics and Crop Plant Research

^2^ICARDA, International Center for Agricultural Research in the Dry Areas

^3^ International Maize and Wheat Improvement Center

^4^ Synthetic cross obtained from a cross between *T. turgidum* ssp. *durum* with *Aegilops tauschii*

**Table S2. Physico-chemical properties of soil in experimental site (0-60 cm soil depth)**

| **Soil particles (%)** | | | | **Soil texture** | | **EC**  **(ds.m^-1^)** | | **PH** | | **Cu**  **(mg/kg)** | | **Mn**  **(mg/kg)** | | **Fe**  **(mg/kg)** | | **Zn**  **(mg/kg)** | | **P**  **(mg/kg)** | | **K**  **(mg/kg)** | | **T.N**  **(%)** | | **Soil depth**  **(cm)** | | **Year** | |  |
| --- | --- | --- | --- | --- | --- | --- | --- | --- | --- | --- | --- | --- | --- | --- | --- | --- | --- | --- | --- | --- | --- | --- | --- | --- | --- | --- | --- | --- |
|  | | **Sand** | **Clay** | **Silt** | |  | |  | |  | |  | |  | |  | |  | |  | |  | |  | |  | |  |
|  | | 41 | 25 | 34 | | Loam | | 2.1 | | 7.70 | | 1.3 | | 20.1 | | 14.9 | | 2.5 | | 38.6 | | 495 | | 0.08 | | 0-30 | | 2019-2020 |
|  | | 40 | 26 | 34 | | Loam | | 1.6 | | 7.95 | | 0.85 | | 7.5 | | 10.1 | | 0.63 | | 6.7 | | 300 | | 0.03 | | 30-60 | |  |

**Table S3 Combined analysis of variance for measured traits in 35 wheat genotypes from different ploidy and species evaluated under two moisture environments**

**(well-watered and water stress)**

|  |  |  |  |  |  | **Mean squares** |  |  |  |  |  |
| --- | --- | --- | --- | --- | --- | --- | --- | --- | --- | --- | --- |
| **Fe** | **Zn** | **FLW** | **FLL** | **PH** | **KD** | **KL** | **TKW** | **NKS** | **GY** | **DF** | **Source of variation** |
| 42270.59^**^ | 6463.96^**^ | 93.39^**^ | 4630.24^*^ | 1838.79^**^ | 6.07^**^ | 7.45^**^ | 4648.64^**^ | 4247.65^**^ | 1428152^¢^.90^**^ | 1 | Env |
| 216.49 | 3555.07^**^ | 6.54 | 681.26 | 171.81^*^ | 0.01 | 0.05 | 57.72^*^ | 248.72 | 26284.13 | 2 | Rep (Env) |
| 7394.07^**^ | 5256.82^**^ | 37.52^**^ | 6551.58^**^ | 331.52^**^ | 0.01 | 0.38 | 112.19^**^ | 1589.98^*^ | 47530.27^*^ | 3 | App |
| 401.37 | 113.35 | 19.96 | 2789.19^**^ | 95.86 | 0.05 | 0.57^*^ | 36.59 | 1306.75 | 67607.04^**^ | 3 | Env*App |
| 151.38 | 713.37^**^ | 10.07^**^ | 3347.68^**^ | 95.26^*^ | 0.00 | 0.18 | 25.25 | 649.25 | 24551.06 | 6 | Rep* App (Env) |
| 1151.10^**^ | 1075.17^**^ | 151.44^**^ | 27134.46^**^ | 1992.71^**^ | 1.33^**^ | 10.70^**^ | 1582.68^**^ | 6817.85^**^ | 1965183.44^**^ | 34 | Gen |
| 168.44 | 100.47 | 2.97 | 930.30 | 92.88^**^ | 0.05^**^ | 0.42^**^ | 50.08^**^ | 1010.13^**^ | 103567.29^**^ | 34 | Env*Gen |
| 232.64 | 158.44 | 3.75 | 860.53 | 92.39^**^ | 0.02 | 0.20 | 20.90 | 665.60^*^ | 45484.24^**^ | 102 | App*Gen |
| 76.75 | 117.77 | 3.67 | 765.63 | 48.12 | 0.02 | 0.21 | 19.43 | 681.67^*^ | 21838.49^**^ | 102 | Env*App*Gen |
| 737.63^**^ | 353.11^**^ | 250.77^**^ | 46925.98^**^ | 38.38^**^ | 2.22^**^ | 1.38^**^ | 2773.60^**^ | 329.79^**^ | 2081315.54^**^ | 2 | PL |
| 12.83 | 1.40 | 0.42^*^ | 104.68 | 3.80 | 0.05^**^ | 0.10 | 44.21 | 29.16 | 96021.16^**^ | 2 | Env* PL |
| 15.60 | 17.45 | 0.45 | 63.41 | 12.84^**^ | 0.00 | 0.00 | 1.80 | 19.33 | 8495.76^**^ | 6 | App * PL |
| 15.20 | 17.56 | 0.40^*^ | 24.29 | 1.99 | 0.00 | 0.01 | 2.03 | 45.19 | 3198.66 | 6 | Env* App* PL |
| 205.62 | 137.13 | 778.67 | 778.67 | 43.01 | 0.02 | 0.21 | 16.94 | 488.10 | 12328.91 | 272 | Error |
| 16.41 | 17.95 | 14.01 | 14.01 | 5.74 | 5.49 | 6.61 | 12.77 | 21.06 | 16.87 |  | CV (%) |

Moisture environment (Env), Replication (Rep), Application (App), Genotype (Gen), Ploidy level (PL), Coefficient of Variation (CV %), Degree of Freedom (DF), Grain yield (GY g/m^2^), Number of kernel per spike (NKS), Thousand kernel weight (TKW g), Kernel length (KL mm), Kernel diameter (KD mm), Plant height (PH cm), Flag leaf length (FLL mm), Flag leaf width (FLW mm), Grain zinc content (Zn µg/g), Grain iron content (Fe µg/g).

*Note:* **, * indicate significant at 0.01 and 0.05 probability levels, respectively.

**Table S4 Analysis of variance for measured traits in 35 wheat genotypes from different ploidy and species in each moisture environment (well-watered and water stress)**

|  |  |  |  |  |  | **Normal** |  |  |  |  |  |
| --- | --- | --- | --- | --- | --- | --- | --- | --- | --- | --- | --- |
| **Fe** | **Zn** | **FLW** | **FLL** | **PH** | **KD** | **KL** | **TKW** | **NKS** | **GY** | **DF** | **Source of variation** |
| 18.16 | 4586.51 | 12.98 | 23.73 | 186.74 | 0.02 | 0.11 | 115.39 | 416.02 | 19181.44 | 1 | Rep |
| 2695.98^*^ | 2752.53 | 23.28 | 1928.54 | 241.41 | 0.02 | 0.26^*^ | 116.13^**^ | 614.95 | 55567.56 | 3 | App |
| 273.30 | 671.77 | 17.59 | 1107.04 | 77.05 | 0.01 | 0.02 | 47.60 | 1110.62 | 36311.94 | 3 | Error a |
| 596.12^**^ | 545.68^**^ | 71.47^**^ | 14316.77^**^ | 1161.79^**^ | 0.88^**^ | 6.24^**^ | 989.94^**^ | 2987.80^**^ | 2012670.69^**^ | 34 | Gen |
| 31.43 | 412.96^*^ | 1.36 | 68.27 | 308.50^**^ | 0.06^**^ | 12.97^**^ | 180.67^**^ | 3113.97^*^ | 2398.75^**^ | 5 | 2x |
| 271.15^*^ | 462.34^**^ | 30.96^**^ | 4206.06^**^ | 1560.94^**^ | 0.40^**^ | 4.86^**^ | 329.12^**^ | 3351.86^**^ | 902451.50^**^ | 18 | 4x |
| 915.33^**^ | 569.01^**^ | 20.67^**^ | 2788.50^**^ | 1037.46^**^ | 0.09^**^ | 4.80^**^ | 181.70^**^ | 2514.65^**^ | 872025.75^**^ | 9 | 6x |
| 1001.13^**^ | 236.12^**^ | 164.72^**^ | 180865.18^**^ | 315.57 | 198.99^*^ | 315.57 | 7643.78^**^ | 2395.82 | 4907050.44^**^ | 2 | PL |
| 146.98 | 141.94^**^ | 3.14 | 748.88 | 61.66^*^ | 0.02 | 0.11 | 27.59^*^ | 574.14 | 50654.06^**^ | 102 | App*Gen |
| 327.55 | 3.49 | 0.99 | 242.27 | 87.48 | 12.97 | 87.48 | 10.90 | 551.41 | 13814.50 | 6 | App*PL |
| 157.42 | 114.89 | 1.06 | 326.07 | 43.78 | 0.03^*^ | 0.23 | 7.46^**^ | 525.12 | 1813.38 | 15 | App*2x |
| 133.74 | 170.27 | 3.08 | 1041.11 | 63.47^*^ | 0.01 | 0.11 | 32.54 | 517.19 | 46638.25^**^ | 54 | App*4x |
| 181.63 | 93.98 | 3.52 | 419.57 | 67.51 | 0.01 | 0.05 | 28.76 | 725.63 | 82609.50^**^ | 27 | App*6x |
| 193.25 | 126.52 | 2.68 | 829.03 | 45.26 | 0.02 | 0.13 | 19.25 | 641.42 | 17824.25 | 136 | Error b |
| 17.67 | 18.19 | 14.26 | 14.67 | 5.79 | 5.01 | 5.09 | 12.49 | 23.53 | 16.26 |  | CV (%) |
|  |  |  |  |  | **Water-stress** |  |  |  |  |  |  |
| **Fe** | **Zn** | **FLW** | **FLL** | **PH** | **KD** | **KL** | **TKW** | **NKS** | **GY** |  |  |
| 414.82^*^ | 2523.63 | 0.10 | 1338.79 | 156.89 | 0.01 | 0.01 | 0.05 | 81.43 | 33386.75 | 1 | Rep |
| 5099.47^**^ | 2617.64 | 34.20^*^ | 7412.24 | 185.97 | 0.03^*^ | 0.68 | 32.66^*^ | 2281.77^*^ | 59569.69 | 3 | App |
| 29.46 | 754.97 | 2.56 | 5588.35 | 113.47 | 0.01 | 0.35 | 2.90 | 187.87 | 12790.13 | 3 | Error a |
| 23.43^**^ | 629.96^**^ | 82.95^**^ | 13747.98^**^ | 923.80^**^ | 0.51^**^ | 4.88^**^ | 642.82^**^ | 4840.18^**^ | 681080^**^ | 34 | Gen |
| 82.57 | 772.30^*^ | 5.12^**^ | 1017.48 | 127.69^*^ | 0.02 | 13.43^**^ | 166.65^**^ | 3650.87^**^ | 4091.06^**^ | 5 | 2x |
| 547.50^**^ | 436.18^**^ | 37.98^**^ | 3862.19^**^ | 1250.57^**^ | 0.22^**^ | 2.48^**^ | 211.44^**^ | 6016.79^**^ | 450806.25^**^ | 18 | 4x |
| 937.95^**^ | 801.23^**^ | 20.18^**^ | 3454.91^**^ | 847.70^**^ | 0.16^**^ | 3.74^**^ | 214.34^**^ | 3690.89^**^ | 597376.88^**^ | 9 | 6x |
| 896.53^**^ | 303.25^**^ | 839.76^**^ | 192893.27^**^ | 158.83 | 234.53^*^ | 158.83 | 12598.60^**^ | 1471.24 | 11580329.38 | 2 | PL |
| 162.41 | 134.27 | 4.27 | 877.28 | 79.85^**^ | 0.03 | 0.30 | 12.73 | 773.13^**^ | 16668.56 | 102 | App*Gen |
| 167.33 | 2.83 | 6.53 | 627.21 | 60.73 | 19.61 | 60.73 | 28.41 | 554.50 | 81271.44 | 6 | App*PL |
| 63.56 | 91.73 | 2.66 | 596.21 | 58.36 | 0.04 | 0.34 | 6.42 | 695.62 | 1493.56 | 15 | App*2x |
| 146.17 | 136.45 | 4.83 | 1020.38 | 93.08^**^ | 0.03 | 0.37 | 15.92 | 766.94^**^ | 17566.69^**^ | 54 | App*4x |
| 236.35 | 143.27 | 4.77 | 888.32 | 63.64^*^ | 0.02 | 0.20^*^ | 10.28 | 877.84^**^ | 23937.13^*^ | 27 | App*6x |
| 217.99 | 147.57 | 3.52 | 728.30 | 40.76 | 0.02 | 0.29 | 14.63 | 334.72 | 6833.56 | 136 | Error b |
| 15.37 | 17.71 | 15.24 | 13.36 | 5.67 | 6 | 7.91 | 13.03 | 17.91 | 16.69 |  | CV (%) |

Replication (Rep), Application (App), Genotype (Gen), Ploidy level (PL), Coefficient of variation (CV), Degree of freedom (DF), Grain yield (GY g/m^2^), Number of kernel per spike (NKS), Thousand kernel weight (TKW g), Kernel length (KL mm), Kernel diameter (KD mm), Plant height (PH cm), Flag leaf length (FLL mm), Flag leaf width (FLW mm), Grain zinc content (Zn µg/g), Grain iron content (Fe µg/g).

Note: **, * indicate significant at 0.01 and 0.05 probability levels, respectively.
